# Six Weeks of Polarized Versus Moderate Intensity Distribution: A Pilot Intervention Study

**DOI:** 10.3389/fphys.2020.534688

**Published:** 2020-11-12

**Authors:** Golo Röhrken, Steffen Held, Lars Donath

**Affiliations:** Department of Intervention Research in Exercise Training, German Sport University, Cologne, Germany

**Keywords:** polarized training, HIIT, polarization index, endurance training, triathlon, overtraining, TID, moderately trained

## Abstract

**Background:**

Previous research indicates that polarized training-intensity-distribution (TID) programs could enhance endurance performance. Short-distance triathletes, however, perform most of their competition-specific training around moderate-intensity intervals. There is still a lack of evidence as to which program is more beneficial during triathlete training. This pilot study examined 6 weeks of training-macrocycle using polarized intensity distribution compared to moderate TID and it’s effects on sub-maximal and maximal performance indices during running and cycling.

**Methods:**

Fifteen moderately trained triathletes were either assigned to an intervention group (INT, *n* = 7, 2 females/5 males, Age: 29.1 ± 7.6) or a control group (CON, *n* = 8, 2 females/6 males, Age: 30.3 ± 6.1). We used the minimization method (Strata: gender, age competition times, training volumes) to allocate the groups. The participants underwent incremental cycling and running testings before and after the intervention period to assess performance indices until objective exhaustion. CON employed a moderate TID with either medium-intensity (MIT) or low-intensity training (LIT). INT used polarized training intensity distribution (TID), with either LIT or high-intensity training (HIT). Average training hours and anthropometric data did not indicate any differences between CON and INT during the study period. We applied the polarization index of >2 in INT (2.1 ± 0.4) and <1 in CON (0.9 ± 0.3).

**Results:**

Both groups notably improved their lactate threshold 2 (+2.8 ± 5.1 %, *p* = 0.026) and peak (+5.4 ± 6.2 %, *p* = 0.002) running performance. We did not observe statistically significant time × group interaction effects in any of the performance outcomes between both groups.

**Conclusion:**

Polarized TID in moderately trained triathletes did not prove to be superior compared to a more moderate TID. However, more studies in larger and more highly trained subjects are needed.

## Introduction

Adequate training scheduling including traditional, undulating, or block periodization is of paramount importance for performance peaking in endurance athletes (Bompa and Haff, 2009; Issurin, 2016). Studies reveal that the right balance between low-intensity training (LIT) and high-intensity training (HIT) sessions over a 6–12 week period can improve performance compared to sessions limited to either low intensity or high intensity (Laursen, 2010).

In a short-distance triathlon, most of the race intensity is close to or slightly below the second lactate threshold (LT2) (Zhou et al., 1997). In a long-distance triathlon, the race intensity is closer to or slightly below your first lactate threshold (LT1) (Muñoz et al., 2014). Moderate-intensity sessions comprise competition-specific intensity levels in a short-distance triathlon. Key interval-sessions are often around LT2 intensity levels.

Consensus about race intensity in a triathlon is lacking because some parts of the race are performed close to intensity above LT2 intensity (Hausswirth and Brisswalter, 2008; Cejuela, 2009). In polarized TID, athletes spent approximately 75–90% of their training volume below the LT1, 0–5% between LT1 and LT2, and 5–20% at intensities above LT2 (Treff et al., 2019). These findings indicate that the TID in triathlon-training is often more moderate or LT2-based, and not polarized (Muñoz et al., 2014).

HIT increases cardiac stroke volume and activates the molecular PGC-1alpha pathway that induces mitochondrial biogenesis (Gibala, 2009; Hawley et al., 2014). A polarized TID results in favorable adaptations, mainly by prioritizing HIT over moderate-intensity training (MIT) sessions (Seiler and Kjerland, 2006; Egan and Zierath, 2013). Studies show performance-enhancing effects of a polarized TID compared to moderate based TIDs in endurance athletes (Seiler and Kjerland, 2006). In this regard, a polarized TID expands time to exhaustion and increases V?O_2*max*_ in highly trained endurance athletes, compared to moderate TID or only HIT training (Stöggl and Sperlich, 2014). Predominant time spent on MIT is associated with inferior performance increases in moderately trained long-distance triathletes compared to training time in LIT (Muñoz et al., 2014).

However, most studies reported TID in very intense endurance sports, such as cycling, cross-country skiing, and rowing. In these sports, critical parts of the race are done in an intensity close to V?O_2*max*_; similar to the targeted-intensity of a HIT session (Buchheit and Laursen, 2013; MacInnis and Gibala, 2017). Furthermore, some elite athletes’ training diaries show moderate TIDs in a long-term analysis, especially in running (Esteve-Lanao et al., 2005; Plews and Laursen, 2017). In addition, in a recent randomized trial, a polarized TID is not more effective compared to pyramidal TID in the training of highly trained rowers (Treff et al., 2017).

It is not clear whether a polarized TID elicits favorable effects in short-distance triathlon performance. Therefore, this pilot study investigates whether a polarized TID leads to favorable performance benefits compared to a more traditional, moderate-intensity TID in moderately trained triathletes.

## Materials and Methods

### Recruitment and Randomization

Recruitment started in May 2019 by using flyers and handouts at triathlon events in Northern Germany. Participants were informed about the study on social media; thirty-five participants who fulfilled the inclusion criteria were enrolled. Participants had been competitive triathletes for two or more years, and had competed in more than three short-distance triathlons per year. Moreover, to fulfill the inclusion criteria, participants had to be younger than 55 years old. We also used competition data for training-intensity calculation via heart-rate sampling. Thereby, one short-distance triathlon was allowed during the training phase. We tried to keep testing at the same time of the day for pre and post testing. Athletes tapered for 7 ± 2 days prior testing, with only 30–60 min of low-intensity training and some motoric activation (4 × 6 s sprints).

Participants were allocated into an intervention group (INT) or a control group (CON). We used the minimization method to randomly allocate athletes to INT and CON by their gender, age, training volume, and a predefined fitness index. The Fitness index is a calculation based on running times in a 10 km event [10 k (min.)], and 95% of cycling power in a 20-min test [CP20 (W)] (see Table 1).

**TABLE 1:** Participants’ anthropometric and fitness characteristics before the study.

	INT (*n* = 7)	CON (*n* = 8)	*p*	SMD**
Gender (f/m)	2/5	2/6	0.887	0.08
Age (years)	29.1 ± 7.6	30.3 ± 6.1	0.763	0.16
Weight (kg)	76.4 ± 9.55	73.5 ± 7.36	0.304	0.55
Bike 20 min. best (W/kg)	3.6 ± 0.64	3.7 ± 0.53	0.961	0.02
Run 10 km best (min)	39.3 ± 5.8	39.1 ± 6.1	0.949	0.03
Prior training volume (h/wk)	11.7 ± 1.6	12.0 ± 2.51	0.580	0.57

Values are mean ± Standard Deviation (SD). NT, Intervention; CON, Control Group. **Calculated via Cohen’s d in a t-test for each group.

The formula to calculate the fitness index is: $\frac{CP20\left(W\right)}{\left(50/10\text{k}\left(\min.\right)\right)}$

### Participants

All participants were moderately trained triathletes, competing in various regional short-distance triathlon events in Northern Germany with an average training volume of 11 h 54 min ± 1 h 54 min of endurance training in swimming, cycling, and running per week at baseline testing. A short-distance triathlon event consists of 750–1,500 m of swimming, 20–40 km of cycling, and finishes with 5–10 km of running. Athletes were at the end of their competitive season with one or two triathlons remaining.

Athletes performed 3.62 W/Kg (±0.62 W) at LT2 and 362 Watts (±62 Watts) at Peak-Power-Output. Athletes are moderately trained; according to the criteria of Pauw et al. (2013) for cycling related assessment of performance. Some athletes reported athletic and strength training before the study and were told to maintain the same training regimen in addition to their program. We recorded 6 weeks of triathlon training data. We did not observe any differences in anthropometrical or performance data between CON and INT before the intervention-period (see Table 1). Athletes withdrew from the study if they missed more than three training sessions in 1 week, or if their overall volume of training was below 20% of the prescribed program. Three athletes of INT and two athletes of CON needed to be excluded from the study due to sickness, other health impairments, or personal reasons.

### Design

This intervention trial is a non-blinded, single-center randomized controlled trial. A parallel-group design compared 6 weeks of endurance exercise training based on a polarized TID to a more moderate TID. Primary and secondary outcome parameters were assessed at baseline (t0) and after the intervention (t1). The study protocol complied with the Declaration of Helsinki, and the Ethical Committee of the German Sport University approved the study (130/2019). Participants provided informed consent after receiving all relevant study information.

### Exercise Testing

**FIGURE 1:**
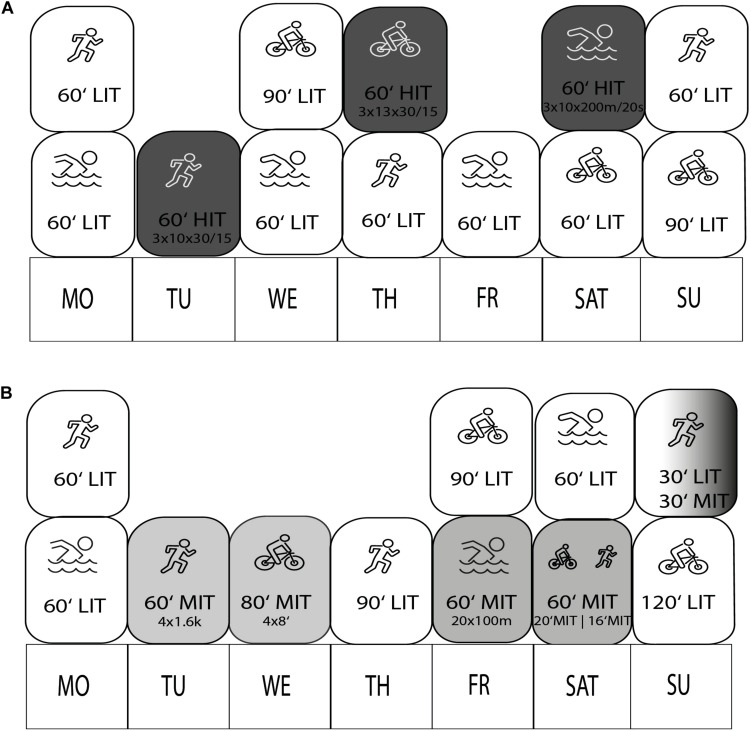
Weekly training schedule of INT **(A)** and CON **(B)** during the 6 weeks study period. LIT, low intensity training; MIT, medium intensity training; HIT, high intensity training.

Before (t0) and after (t1) of the intervention, each athlete took part in a 1-day test procedure to evaluate LT1 and LT2 as well as maximum values for running and cycling (see Figure 1). We measured lactate values in the last minute of every stage (see below). After exercise testing, we used the standard-average-mean of two methods to calculate LT1 and LT2. Method 1 used a third grade polynomic fitting of power and lactate data to calculate two and four mmol/l values (Westhoff et al., 2013). Method 2 used the minimal-lactate-equivalent method for LT1 and minimal-lactate-equivalent +1.5 mmol/L for LT2 (Faude et al., 2009).

Within the test procedure, we calculated LT1 and LT2 as well as the corresponding values for power and heart rate. A swimming test was not used due to restricted access and swimming pool restrictions.

The incremental stage tests for cycling and running consisted of a relaxed warm up of 10 min at 100 W or 8 km/h. Athletes then started with the first 3-min stage at 120 W for females and 150 W for males or at 10 km/h for running on the treadmill. After every 3 min, there was a 30 W/+1.5 km/h increment in resistance for men and a 25 W/+1.5 km/h increment for women. In the final 30 s of each stage, heart rate, lactate, and rate of perceived exhaustion (RPE, 1–10) (Foster et al., 2001) were recorded. To perform the lactate analysis, an exercise physiologist drew 20 μL of capillary blood from the hyperemic earlobe, and the level of blood lactate was analyzed [Accutrend, Roche, Basel (Switzerland)]. The cycling test stopped when lactate levels reached + 3.5 mmol/l compared to the resting level. Athletes then started with a 10-min active-recovery period at 100 W and began with the running test.

**FIGURE 2:**
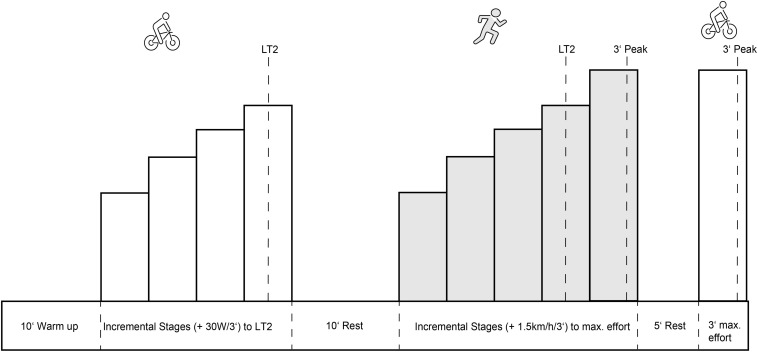
Exercise test design: Participants did a sub-maximal incremental stages test (+25–30W/3’) on the bike and a maximal incremental stages-test (+1.5 km/h/3’) on the treadmill. Participants finished the test procedure with a maximal 3’ effort on the bike (Peak). Included in the test procedure was a 10’ warm-up, 10’ rest between the incremental stages-tests and a 5’ rest period before the final all-out test. LT, lactate threshold; Peak, peak performance.

After a 10-min warm up on the treadmill, athletes started with the first stage and stopped after every stage at the treadmill’s side panel to allow for blood sampling. They then returned immediately to running after each sample was taken from the earlobe. Athletes repeated the stages until they objectively exhausted. The sampling time between each running stage was below 30 s. We determined physical exhaustion criteria strictly in line with the requirements of the study of Midgley and coworkers for the determination of V?O_2*max*_ plateaus (Midgley et al., 2007). Cycling and running test participants reached exhaustion when two out of four considered levels were completed. The exhaustion levels are ≥91% of the age-predicted maximum heart rate (Tanaka et al., 2001), the exponential rise of lactate ≥5.6 mmol/l (Faude et al., 2009), and the RPE values ≥7 (Foster et al., 2001). After maximum exhaustion in the final running stage, athletes rested for 5 min and finished the testing with a 3-min cycling all-out test on the ergometer (see Figure 2).

Participants used their own bike, controlled by an ergometer, during the cycling test (Wahoo Kickr, Atlanta, GA, United States) and did the running test on a treadmill (Woodway, Michigan, MI, United States). To avoid data deviation, participants used their own power-meter, calibrated before the testing, to determine training-zones and interval-intensity. Athletes uploaded and stored their training data in a web application (Trainingpeaks, Boulder, United States). Furthermore, athletes calibrated their power meter before every training session to limit variances between the equipment. Before the study began, an exercise physiologist conducted an informative interview to familiarize athletes with the testing and training procedures.

### Training Intensity Distribution and Calculation

The present study used a heart rate in zones approach since this method reflects the internal physiological strain of each training session (Sylta et al., 2014). For the training load calculation, we analyzed heart rate via a training impulse model (TRIMP/wk) (Lucia et al., 2003). Furthermore, to quantify the training intensity, we used a model based on the three-zone classification system by the Norwegian Sports Federation (Tønnessen et al., 2014).

Three-Zone Model Corresponding to power, pace, heart rate, rate of perceived effort (RPE).

1. Z1: LIT, Pace/Power/Heart rate below LT1, RPE ≤ 4
2. Z2: MIT, Pace/Power/Heart rate between LT1 and LT2, RPE > 4, < 7
3. Z3: HIT, Pace/Power/Heart rate above LT2, RPE ≥ 7

In the first step, we calculated TID via the heart-rate time-in-zone approach. The time-in-zone method summarizes the time and then calculates each intensity zone’s percentage corresponding to certain lactate thresholds and peak values. In a second step, if heart-rate values were missing, data were complemented by external parameters (e.g., power, pace). In a third step, the average session rate of perceived exhaustion (sRPE) for each swim session was monitored and also added to the TID calculation. sRPE was quantified by a one (no exhaustion) to ten (high level of exhaustion) scale, described by studies from Foster and colleagues (Foster et al., 2001). These data went into the “Polarization-Index” equation by Treff et al. (2019), calculated as follows: $\log10\left(\frac{Z1}{Z2\times Z3\times 100}\right)$ (Treff et al., 2019).

### Training Intervention

The training intervention period for INT and CON ran over 6 weeks of similar duration and frequencies of weekly training sessions (see Figure 1). Participants performed all sessions using their equipment, training environments, and facilities. Table 1 depicts an overview of both groups’ relevant descriptive training parameters (INT and CON) during the intervention. Athletes training 13 h per week CON had a slightly higher prescribed training load with 941 TRIMP/wk to 788 TRIMP/wk in CON (see Table 2); when assessing training load via TRIMP/wk (Lucia et al., 2003).

**TABLE 2:** Completed training volume, load, and time-in-zones during the study period for Intervention and Control group.

	INT (*n* = 7)	CON (*n* = 8)	*p*	SMD**
**Prescribed**				
Training-Sessions per week	9 × LIT, 3 × HIT	6 × LIT, 6 × MIT		
TID (%LIT/%MIT/%HIT)	92/0/8	65/35/0		
∑ Training load (TRIMP/wk)	788	941	0.352	0.50
Polarization Index (a.U.)	>2.0	<1.0		
**Completed**				
Training Volume (h/wk)	10.8 ± 2.4	10.0 ± 2.7	0.580	0.29
Z1, LIT Volume (%)	75.2 ± 14.4	77.8 ± 11.9	0.704	0.20
Z2, MIT Volume (%)	11.1 ± 10.9	20.3 ± 10.8	0.118	0.86
Z3, HIT Volume (%)	13.7 ± 4.1	2.0 ± 1.5	<0.001	3.84
Polarization Index (a.U.)	2.1 ± 0.4	0.9 ± 0.3	<0.001	3.88
∑ Training Load (TRIMP/wk)	882.0 ± 155	739.0 ± 162	0.106	0.90
Load in LIT (TRIMP/wk)	499.0 ± 171.0	480.0 ± 174.0	0.832	0.11
Load in MIT (TRIMP/wk)	126.0 ± 104.0	223.0 ± 82.0	0.064	1.05
Load in HIT (TRIMP/wk)	257.0 ± 65.7	35.9 ± 24.8	<0.001	4.58
Completed to Prescribed Load (%)	112.0 ± 14.9	86.2 ± 10.0	0.001	2.10

Values are mean ± Standard Deviation (SD). INT, Intervention; CON, Control Group; a.U., arbitrary unit. **Calculated via Cohen’s d in a t-test for each group.

In INT, training consisted of HIT and LIT Sessions. LIT sessions were between 60 and 90 min and HIT sessions were between 60 and 70 min in duration. Weekly training sessions in INT were three high-intensity (HIT) sessions, one in every discipline, interspersed by five LIT Sessions (see Figure 1). We aimed for a total duration of 92% of the time in LIT (%LIT) and 8% time in HIT (%HIT). These TIDs are in line with studies done on elite cyclist and triathletes (Rønnestad et al., 2016; Selles-Perez et al., 2019), but substantially higher compared to studies done in elite rowers or xc-skiers (Sylta et al., 2014; Treff et al., 2019). We decided to apply a potent adaption stressor in a medium-short intervention period of 6 weeks to achieve a more pronounced intensity in INT. Accordingly, we aimed for a Polarization Index of ≥2.0 for INT and <1.0 for CON.

In CON, training sessions consisted of LIT or MIT sessions. Athletes completed five MIT sessions and four LIT sessions per week. LIT sessions were between 60 and 120 min and MIT sessions were between 60 and 80 min in duration. We prescribed the athletes 65% of total training time in LIT and 35% time in MIT (%MIT) (see Figure 1). Some authors describe this TID as a moderate-intensity or threshold periodization (Treff et al., 2019).

LIT sessions were continuous endurance sessions below LT1, controlled by heart rate and RPE. For swimming, athletes received a table with LIT workouts based on skill and experience, ranging from 2,400 to 4,600 m. Based on RPE levels and muscular tiredness, athletes were encouraged to reduce exercise intensity in all their LIT sessions when feeling tired.

MIT sessions consisted of intervals or continuous sessions between LT1 and LT2. Athletes regulated intensity in the continuous sessions by RPE, but heart rate and pace should be maintained above LT1 and below LT2. Athletes set a heart rate alarm and reduced intensity if the heart rate reached ≥ LT2. MIT Sessions consisted of one progressive run, 4 × 1,600 m Intervals on the track and one 20-min tempo Brick run per week. For the 1,600 m intervals, athletes aimed for a pace between 95 and 98% of LT2 pace with 3 min of active rest. In cycling, athletes performed one 4 × 8 min interval Session at 88–92% of the LT2 Power, with 2 min of active rest between intervals, and one continuous 120-min tempo ride with a “fartlek” intensity regulation between 80 and 90% of LT2 Power. In swimming, athletes performed one MIT session, which consisted of a 20 × 100 m swim in a medium-effort RPE. If athletes knew their 1,500 m pace for a short-distance triathlon, athletes added 2–3 s to their competition splits and maintained this pace throughout the set. We adjusted each MIT session’s interval length for athletes training below 12 h/wk while retaining the general interval structure. The percentage of MIT time per week was 18–25% for all athletes in CON (see Figure 2). Studies with pyramidal or threshold TIDs report of similar distributions of intensity in rowers and triathletes (Plews and Laursen, 2017; Selles-Perez et al., 2019).

HIT sessions were intermittent-exercise of 30 s short-interval work bouts, interspersed with 15 s active-rest as described in cycling studies from Rønnestad et al. showing higher effectiveness in V?O_2*max*_ and maximal power output improvements (Rønnestad and Hansen, 2016). Athletes did intermittent exercise protocols also in running and swimming.

However, swimming intervals were 3 × 10 × 50 meters work bouts with 20 s passive recovery, as continuous swimming would be challenging to manage. We used the “isoeffort” method to achieve similar intensity in all work bouts; the achieved average intensity of the accumulation of work-bouts was set as high as manageable (Sylta et al., 2016). Athletes rested for a minimum of 36 h of recovery or did an LIT session between each HIT interval session, to ensure recovery, and avoid non-, or functional overreaching. We kept the general weekly structure for athletes in INT training <12 weekly training hours. Therefore, maintaining three HIT sessions per week, one in every discipline, but adjusted the interval repetitions of each session (ex. 3 × 10 × 30 s/15 s) to ensure respective 6–8% of total training time in HIT per week.

### Statistics

A Shapiro-Wilk test analyzed the evaluation of the normality distribution before the analysis of the data. Furthermore, we used a student’s *t*-tests to calculate changes in anthropometric data and pre-intervention performance differences between the groups. We used nonparametric calculations (Wilcoxon-Mann-Whitney-Test) and a rank correlation (Spearman) when data were not evenly distributed. Repeated analyses of variances (rANOVA) were used to demonstrate training and performance differences during the intervention of INT and CON by using INT versus CON × 2 (time: t0 versus t1) for power and pace corresponding to LT1, LT2 and peak values. Baseline values were included as a covariate (Vickers and Altman, 2001).

We used effect sizes for variance analyses as partial eta squared with values of ≥0.01, ≥0.06, and ≥0.14 indicating small, moderate, and large effects (Cohen, 1988). For pairwise effect size estimation between groups, standard mean differences (SMD) were additionally calculated as differences between group separately divided by the pooled standard deviations of both groups (trivial: SMD < 0.35, small: 0.35 ≤ SMD < 0.8, moderate: 0.8 ≤ SMD < 1.5, large SMD ≥ 1.5) (Turner et al., 2015). We used the Pearson’s coefficient for the correlation analysis. The software used in this study for statistical analysis were jamovi (the jamovi project, computer software, Version 1.7) and GraphPad Prism^®^ (version 7.0, GraphPad Software, La Jolla, CA, United States). If not specified, data are presented as means ± standard deviation (SD). A *p*-value below 0.05 was considered statistically significant.

## Results

### Tests of Normality

Data is evenly distributed, except for running t0 LT1 performance (*p* = 0.004) and running t0 Peak performance (*p* = 0.033).

### Training Load and Time-in-Zones

Average training hours per week were not different between INT and CON (see Table 1) and we did not find any performance baseline differences between the group in cycling or running (see Table 2). No Training-load differences reveal between INT and CON. However, the percentage of completed to prescribed Training load was significantly higher in INT than in CON (see Table 2).

%LIT and %MIT did not differ between either groups. %HIT was significantly higher in INT compared to CON. The Polarization Index revealed a higher polarization in INT, compared to CON.

### Performance Results

**FIGURE 3:**
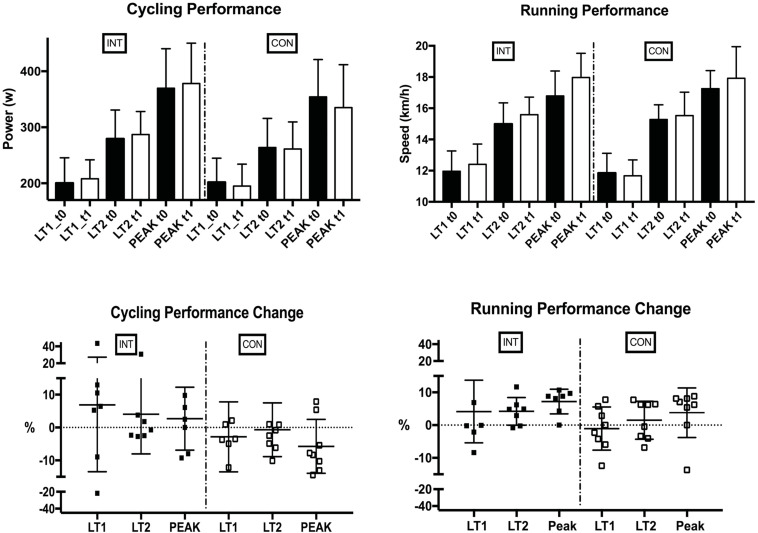
Cycling and running performance, as well as performance differences, during the study period in the Intervention and Control group, displayed as t0 = solid blocks, t1 = transparent blocks. LT, lactate threshold; Peak, peak performance, INT, intervention; CON, control group.

We found small pairwise effect sizes comparing the groups in LT1, LT2 and Peak performance in running and cycling (see Table 3). Neither cycling nor running performance significantly changed from t0 to t1 between INT and CON in any parameter (see Figure 3).

**TABLE 3:** Effect sizes for the differences in the changes between groups and performance change for Intervention and Control group during the study period.

	INT (*n* = 7)	CON (*n* = 8)	SMD**
**Run**			
LT1 (%)	4.2 ± 9.5	−1.1 ± 6.6	0.47
LT2 (%)	4.2 ± 4.2	1.5 ± 5.8	0.50
Peak (%)	7.2 ± 3.8	3.8 ± 7.6	0.65
**Bike**			
LT1 (%)	7.4 ± 19.5	−2.2 ± 10.7	0.71
LT2 (%)	4.5 ± 12.0	−0.7 ± 8.2	0.49
Peak (%)	2.7 ± 9.6	−5.7 ± 8.2	0.89

Values are mean ± Standard Deviation (SD). INT, Intervention; CON, Control Group. **Calculated via Cohen’s d in a t-test for each group.

**FIGURE 4:**
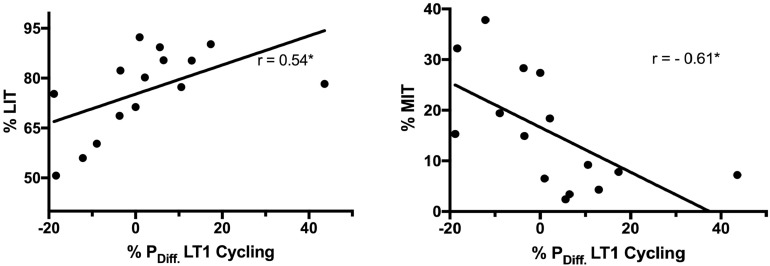
Correlations in all athletes between training time spent in LIT (% LIT) and training time spent in MIT (%MIT) to Change in LT1 cycling performance (% P_*Diff.*_ LT1 Cycling). LT, lactate threshold; LIT, low-intensity training.

**FIGURE 5:**
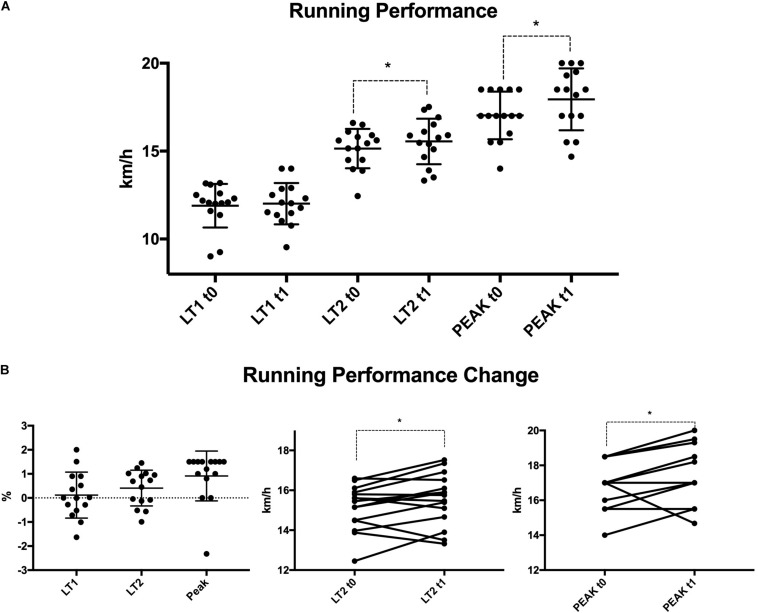
Running performance **(A)** and running performance changes **(B)** in all participants. LT, lactate threshold; Peak, peak performance.

The combined group of participants showed a significant positive correlation between %LIT to cycling LT1 performance changes (*r* = 0.54, *p* = 0.04) and an inverse correlation between %MIT to cycling LT1 performance changes (*r* = −0.61, *p* = 0.009) and %MIT to running Peak performance changes (*r* = −0.52, *p* = 0.049) (see Figure 4). Furthermore, all participants, i.e., taking both groups as one group, improved their Peak and LT2 performance in running (Peak: + 5.4 ± 6.2 % *p* = 0.002 LT2: + 2.8 ± 5.1 % *p* = 0.026) (see Figure 5).

## Discussion

The main finding of this study is that a polarized TID program is not more effective than a traditional, more moderate training program in the training of triathletes. During the 6 weeks intervention, participants in both groups notably improved their Peak and LT2 running performance. We also found small effect sizes when comparing the groups for moderately trained athletes in LT1, LT2 and Peak performance in running and cycling (see Table 3). Furthermore, in both groups, some individuals improved >10%, indicating that both programs might lead to performance benefits. These findings were very prominent in running LT2 and Peak performances (see Figure 5). We did not reveal meaningful group differences, although completed Training-load was significantly higher in INT compared to CON. However, we assume that with a higher sample size polarized TID might elicit superior performance benefits due to medium to high η_*p*_^2^ values in INT (see Table 4).

**TABLE 4:** Running and cycling performance for Intervention and Control group during the study period.

	INT		CON		Group comparison*		
	t0	t1	t0	t1	*F*	*p*	η_p_^2^
**Run**							
Lac max (mmol/L)	7.2 ± 1.5	7.8 ± 1.4	7.5 ± 1.5	7.3 ± 1.5	0.32	0.583	0.02
LT1 (km/h)	12.0 ± 1.3	12.4 ± 1.3	11.8 ± 1.3	11.7 ± 1.0	2.24	0.160	0.16
LT2 (km/h)	15.0 ± 1.4	15.6 ± 1.2	15.3 ± 0.9	15.5 ± 1.5	0.71	0.416	0.06
Peak (km/h)	16.8 ± 1.6	18.0 ± 2.0	17.3 ± 1.2	17.9 ± 2.0	0.96	0.346	0.07
**Bike**							
Lac min (mmol/L)	1.6 ± 0.3	1.7 ± 0.3	1.5 ± 0.4	1.6 ± 0.4	0.02	0.891	0.00
Lac max (mmol/L)	10.6 ± 2.2	9.9 ± 2.3	10.5 ± 2.9	9.2 ± 2.2	0.12	0.728	0.01
LT1 (W)	204 ± 48.0	212 ± 36.0	202 ± 42.0	196 ± 38.0	1.57	0.234	0.12
LT2 (W)	282 ± 54.0	291 ± 43.0	264 ± 52.0	261 ± 48.0	1.86	0.197	0.13
Peak (W)	370 ± 70.0	378 ± 72.0	354 ± 67.0	335 ± 77.0	2.71	0.125	0.18

Values are mean ± Standard Deviation (SD). INT, Intervention; CON, Control Group. *Repeated ANOVA, effect sizes were calculated as partial η_p_^2^.

LIT is of paramount importance during the training-process of endurance athletes (Seiler and Kjerland, 2006). One concept to describe the benefit of LIT is that it balances and stabilizes adaptations resulting from intense training bouts, done by HIT beforehand (Hawley et al., 2014). Furthermore, LIT stabilizes hormonal responses, thereby avoiding non-functional and functional over-reaching in elite and recreational athletes (Hill et al., 2008). An interesting finding of this study is that %LIT correlates with benefits in participants’ LT1 performance. This is in line with studies over the last 30 years in elite and moderately trained athletes, concluding that %LIT of >60–80% is of paramount importance in endurance training (Föhrenbach et al., 1987; Billat, 2001). Furthermore, performance in long-distance triathlon relates to %LIT (Muñoz et al., 2014). Studies indicate that 8–12 h of LIT might be an appropriate amount of time to induce adaptations in trained cyclists (Rønnestad and Hansen, 2018). However, this question remains open in recreationally athletes and moderately trained athletes. Interestingly, in both groups %LIT is above 70% (INT = 75.2 ± 14.4% versus CON = 77.0 ± 11.9%).

However, coaches highlight the importance of implementing MIT sessions for elite athletes (Plews and Laursen, 2017); thereby MIT might be a potent stimulus to induce performance change (Milanoviæ et al., 2015). In this regard, MIT oriented TIDs such as a pyramidal or a threshold program show beneficial effects in the training of recreationally trained individuals (Gormley et al., 2008), and in elite athletes (Esteve-Lanao et al., 2005; Plews and Laursen, 2017). Traditional triathlon training programs consist of immense training volumes and a large percentage of training time spent on MIT, compared to other endurance disciplines, especially in training for a middle or long-distance triathlon (Muñoz et al., 2014; Selles-Perez et al., 2019). Training load in MIT was not different between INT and CON. However, we measured Training load via heart rate; thereby, the cardia response to an intensified stimulus likely underestimates the metabolic work performed (Sylta et al., 2014). We found that the high MIT training load and %MIT in INT is possibly a delayed heart rate response to a HIT session, which is also described in elite rowers (Plews et al., 2014). Most short-distance triathletes do their race-specific intervals close to LT2, as race intensity is predominantly considered as a steady-state effort (Suriano and Bishop, 2010). However, an inverse correlation in all participants between %MIT and cycling performance changes in LT1 display (*r* = −0.61, *p* = 0.02). Collaboratively moderate TIDs, such as threshold or pyramidal models, are often inferior compared to polarized models in rowers, cyclists, and xc-skiers (Stöggl and Sperlich, 2014; Sylta et al., 2016). These results indicate that MIT is not a potent stimulus to induce metabolic and cardiovascular adaptions in moderately trained triathletes, compared to HIT.

HIT is a powerful stimulus in enhancing endurance performance (MacInnis and Gibala, 2017). HIT demands a high ATP turnover and cellular energy depletion with an accumulation of reactive molecules and energy intermediates (Hawley et al., 2014). These metabolites accumulate and activate PGC-1alpha, thereby triggering mitochondrial biogenesis (Chandel, 2015). However, %HIT does not show any correlation to any performance change in INT or in any of the participants.

Another point to mention is that a significant percentage of HIT is a risk factor for adverse training effects, i.e., non-functional and functional overtraining (Meeusen et al., 2013). Athletes in INT completed 13.5 ± 4.4% of training in HIT, which is a substantially high amount, compared to what other studies in moderately trained athletes have implied (Muñoz et al., 2014). Rønnestad and colleagues employed even higher %HIT, but only as an intermittent training block for 1 week, and athletes were elite cyclists (Rønnestad et al., 2016). Additionally, athletes in INT completed 112.0 ± 14.9% of the prescribed training load. Non-functional overreaching and maladaptations are associated with high and overly demanding Training-loads and intensified programs (Meeusen et al., 2013). In this regard, two athletes in INT had to withdraw from the intervention due to muscular fatigue and the onset of illness symptoms. Although both groups completed the same training load, we might speculate that the Training-load done in INT is too demanding for most moderate triathletes in training.

There are limitations to the current study. One is the high dropout rate (INT *n* = 3, CON *n* = 1) of athletes, which reduces the sample size and impairs the study power. However, significant changes underpin meaningful results. Another limitation is the absence of a swimming test, as the pool times were very restricted and limited during the time of our study. A further point is %MIT, which is high in the INT group compared to other recent studies (Treff et al., 2017). However, a time-in-zone method displays delayed heart-rate elevations and underreports time in HIT compared to a sessions-goal method (Sylta et al., 2014). The short intervention period (6 weeks) might also be seen as a drawback. However, performance benefits of specific intensified training programs are associated with interventions that are shorter than 8 weeks and mesocycles of comparable length (Billat, 2001; Rønnestad et al., 2016). One further limitation is that cardio-respiratory indices such as V?O_2*max*_ and economy parameters were not recorded. However, the observed pace and power improvements corresponding to LT2 and maximum values are equally related to triathlon performance (Bentley et al., 2008; Suriano and Bishop, 2010).

### Practical Implications

A polarized TID may be superior to traditional, more moderate TID in training triathletes. However, both programs lead to performance improvement in this study. A polarized TID does not result in any beneficial performance benefit and applying an intensified program should be done with caution. Moreover, a high dropout rate in INT indicates that 2–3 HIT sessions per week, although done in different disciplines, accumulate to a substantial stressor on the body.

It is necessary to carefully monitor the Training-load, density, and training-adaptation in any training program. Future studies should concentrate on TID guidelines, TID calculations, and different TID approaches in each sporting discipline. It remains to be revealed whether TID in multisports such as triathlon might differ from TID in “traditional” endurance sports like cycling and rowing.

## Conclusion

In conclusion, a “polarized” TID did not prove beneficial than a moderate TID. Both 6 week TID programs improved performance; neither proved more effective in cycling- or running-training of moderately trained triathletes. However, small group size effects in favor of a polarized TID revealed. On average, all participants, i.e., when combining both groups, improved performance in LT1 and peak running performance. We found correlations in all participants in cycling LT1 performance to training time in LIT and an inverse correlation to training time in MIT.

## Data Availability Statement

The raw data supporting the conclusions of this article will be made available by the authors, without undue reservation, to any qualified researcher. Requests to access the datasets should be directed to GR, golo.roehrken@gmail.com.

## Ethics Statement

The studies involving human participants were reviewed and approved by Ethics Board of the German Sport University, Cologne, Germany. The patients/participants provided their written informed consent to participate in this study.

## Author Contributions

GR planned and designed the study, conducted measurements, performed the statistical analysis, and prepared the manuscript. SH planned and designed the study, and edited the manuscript. LD planned and designed the study, organized the database, and edited the manuscript. All authors contributed to the manuscript revision and approved the submitted version.

## Conflict of Interest

The authors declare that the research was conducted in the absence of any commercial or financial relationships that could be construed as a potential conflict of interest.
